# Intraoperative Radiation Therapy: A Critical Analysis of the ELIOT and TARGIT Trials. Part 1—ELIOT

**DOI:** 10.1245/s10434-014-3998-6

**Published:** 2014-08-27

**Authors:** Melvin J. Silverstein, Gerd Fastner, Sergio Maluta, Roland Reitsamer, Donald A. Goer, Frank Vicini, David Wazer

**Affiliations:** 1 Breast Center, Hoag Memorial Hospital Presbyterian, Newport Beach, CA USA; 2 Department of Surgery, Keck School of Medicine, University of Southern California, Los Angeles, CA USA; 3 Radiotherapy and Radio-Oncology Landeskrankenhaus, Paracelsus Medical University, Salzburg, Austria; 4 Radiation Oncology, Oncological Hyperthermia Unit—Medical Center, Verona, Italy; 5 Department of Senology, Paracelsus Medical University Salzburg, Salzburg, Austria; 6 Physics, IntraOp Medical, Sunnyvale, CA USA; 7 Radiation Oncology, St. Joseph Mercy Oakland, Pontiac, MI USA; 8 Radiation Oncology, Tufts University School of Medicine, Boston, MA USA; 9 Radiation Oncology, Albert Medical School of Brown University, Providence, RI USA

## Abstract

**Introduction:**

Two randomized intraoperative radiation therapy trials for early-stage breast cancer were recently published. The ELIOT Trial used electrons (IOERT), and the TARGIT-A Trial Update used 50-kV X-rays (IORT). These studies were compared for similarities and differences. The results were analyzed and used to determine which patients might be suitable for single-dose treatment.

**Method:**

The primary sources of data were the ELIOT Trial and TARGIT-A Trial, as well as a comprehensive analysis of the peer-reviewed literature of accelerated partial breast irradiation (APBI) using 50-kV X-rays or electrons. Studies published or presented prior to March 2014 were analyzed for efficacy, patient restrictions, complications, and outcome.

**Results:**

With a median follow-up of 5.8 years, the 5-year recurrence rates for ELIOT versus external beam radiation therapy (EBRT) patients were 4.4 % and 0.4 %, respectively, *p* = .0001. A low-risk ELIOT group was identified with a 5-year recurrence rate of 1.5 %. With a median follow-up of 29 months, the 5-year recurrence rates for the TARGIT-A versus EBRT patients were 3.3 % and 1.3 %, respectively, *p* = .042.

**Conclusion:**

With 5.8 years of median follow-up, IOERT appears to have a subset of low-risk women for whom IOERT is acceptable. With 29 months of median follow-up the results of IORT with 50-kV devices are promising, but longer follow-up data are required. At the current time, single-fraction IOERT or IORT patients should be treated under strict institutional protocols.

When breast conserving surgery (BCS) is chosen, excision is commonly followed by 5 weeks of whole breast irradiation (WBI), with or without a boost to the tumor bed. Long radiation schedules are a burden for many women.^1^,^2^ This has stimulated an interest in accelerated partial breast irradiation (APBI) that can reduce overall treatment time without compromising oncological outcomes or cosmesis.^3^,^4^ Intraoperative radiation therapy (IORT) is an attractive APBI approach because it delivers the entire radiation treatment during surgery. Two randomized IORT-APBI trials, ELIOT using electrons and TARGIT-A using 50-kV X-rays, have studied whether IORT can produce results that are equivalent to standard treatment.^5^–^7^ In a series of 2 reports, we analyze these studies to determine whether IORT is ready for incorporation into standard practice and to determine what patient cohorts might be suitable for single-dose treatment.

## Methods

The primary sources of data for these analyses were the ELIOT Trial and TARGIT-A Trials, as well as a comprehensive review of peer-reviewed literature of APBI studies using 50-kV X-rays or electrons, involving 50 or more patients with a minimum of 30 months median follow-up.^5^–^7^

Since the energy source for intraoperative radiation therapy and the technique used for delivery is different for ELIOT and TARGIT-A, each study is discussed in a separate report. The Results Section for each trial summarizes outcomes reported in the trial publications. The Discussion Section uses other studies as well as the trial publications to assess efficacy of the treatment and provide guidance on their use in non-trial environments. Intraoperative radiation therapy given with electrons (ELIOT) is referred to as IOERT. Intraoperative radiation therapy given with 50-kV x-rays (TARGIT A) is referred to as IORT.

## Eliot Trial

### Overview

The ELIOT Trial randomized 1,305 patients, 48 years or older, with tumors 2.5 cm or smaller to either a single dose of 21 Gy prescribed to the 90 % depth or to 50 Gy of external beam radiation therapy (EBRT) and a 10-Gy boost delivered over 6 weeks.^5^ With a median follow-up of 5.8 years, the 5-year recurrence rate was 4.4 % for ELIOT versus 0.4 % for the EBRT (*p* < .0001). The data are summarized in Table 1.

**Table 1:** Oncological events as reported in the ELIOT Trial

	EBRT (*n* = 654)		ELIOT (*n* = 651)		*p* value
	No.	5-year rate	No.	5-year rate	
IBTR	4	0.4 %	35	4.4 %	<.0001
Local (“true”)	4	0.4 %	21	2.5 %	.0003
Elsewhere	0	0	14	1.9 %	.0001
Axillary/regional	2	0.3 %	9	1.0 %	.03
Contralateral breast cancer	13	1.7 %	8	1.1 %	.34
Distant metastases	35	4.8 %	33^a^	5.1 %	.94
Other primary cancer	22	3.2 %	20	2.5 %	.88
Deaths (total)	31	3.1 %	34	3.2 %	.59
Breast cancer	20	2.0 %	23	2.1 %	.56
Other	11	1.1 %	11	1.1 %	.94

Adapted from Table 2, Lancet Oncology^5^

^a^4 IOERT patients diagnosed with metastases at the time of surgery

### Technique 8

After tumor excision, the breast tissue was mobilized. The chest wall and underlying structures were protected with a lead/aluminum shield. The breast tissue to be irradiated was reapproximated over the shield. An appropriately sized collimator (4–8 cm) was inserted. Radiotherapy was performed using a linear accelerator; 21 Gy, to the 90 % isodose, was delivered to the tumor bed.

### Complications

Compared with the conventional arm, ELIOT reported less skin damage (i.e., erythema, dryness, hyper-pigmentation, or itching), *p* = .0002, and no differences for fibrosis, retraction, pain or burning, but a higher incidence of radiologically determined fat necrosis, 5 %, versus 2 %, *p* = .04. In addition, ELIOT had less pulmonary toxicity than the EBRT as diagnosed by follow-up spiral CT (4 in the ELIOT arm and 38 in the EBRT arm). These differences in skin and pulmonary toxicity are not unexpected given the differences in IOERT versus EBRT breast irradiation techniques.

### Local Recurrences

The 5-year ipsilateral breast tumor recurrence (IBTR) rates exceeded 10 % for patients with tumors >2 cm (10 of 83, 10.9 %), 4 or more positive nodes (4 of 31, 15.0 %), poorly differentiated tumors, i.e., grade 3 (15 of 129, 11.9 %), estrogen receptor negative tumors (8 of 63, 14.9 %), or triple negative disease (7 of 43, 18.9 %). Patients with a high proliferative index, i.e., Ki-67 > 20 %, trended to a high IBTR rate (22 of 244, 9.1 %) but did not reach the 10 % threshold. The 5-year IBTR was 11.3 % for the 199 women (30.6 %) with 1 or more of these risk factors vs 1.5 % for the 452 women (69.4 %) with none of these factors (ELIOT Low Risk). The per-protocol results were similar to the intent-to-treat analysis. The IBTR was 4.7 % versus 0.5 % for ELIOT versus EBRT; the 5-year IBTR was 11.8 % for the 178 women (30.4 %) with 1 or more risk factors versus 1.7 % for the 407 ELIOT Low Risk women (69.6 %).

### Regional Failures

Greater regional failure with ELIOT (9 patients, 1.0 %) versus EBRT (2 patients, 0.3 %), *p* = .03, raised concern that fewer regional recurrences with EBRT might be partially due to lower axillary coverage by the tangential breast irradiation.

### Patients with 4 or More Positive Nodes

A total of 69 patients with 4 or more positive nodes received additional EBRT of 50 Gy to the axilla. Those randomized to EBRT received axillary irradiation concurrently. Axillary irradiation was delayed 6–12 weeks for IOERT patients. Timing of adjuvant chemotherapy administration for these patients was not specified.

### Elsewhere Recurrences

There were 14 (1.9 %) versus no (zero) ipsilateral “elsewhere” recurrences with ELIOT vs EBRT, *p* < .0001.

### Contralateral Breast Cancer

There was a nonsignificant higher contralateral breast cancer rate in the EBRT group vs ELIOT (13 vs 8 patients).

### Metastatic Breast Cancer

Metastases, other primary cancers, breast cancer death, and other deaths were similar in the 2 groups (Table 1).

### Survival

Overall survival at 5 years was identical, 96.8 % for the ELIOT group vs 96.9 % for the EBRT group. The 10-year survival remained similar (89.8 % for ELIOT and 92.0 % for EBRT patients).

## Discussion

The ELIOT trial closed in December 2007. Analysis of the results began 5 years after accrual of the last patient. This is important since the time to local recurrence after radiation therapy combined with adjuvant treatment can be delayed.^9^,^10^ Overall, ELIOT patients had a higher 5-year recurrence rate than EBRT patients (4.4 % vs 0.4 %, *p* = .0001). However, ELIOT patients can be divided into low- and high-risk groups based on tumor size, receptor status, nodal positivity, and grade. ELIOT Low-Risk women (69.4 % of the ELIOT patients) had a 5-year IBTR rate of only 1.5 % compared with 11.3 % for the 30.6 % of ELIOT patients with 1 or more high-risk factors.

The American Society of Therapeutic Radiologists (ASTRO) has also published a set of criteria for selecting patients who are suitable for APBI.^11^ For the 23 % of the ELIOT patients who were ASTRO suitable for APBI, the IBTR was 1.5 % at 5 years and equivalent to the IBTR for the EBRT-suitable patients.^12^ The low recurrence for ASTRO-suitable women is consistent with a large series of patients treated with ELIOT off-protocol at the European Institute of Oncology (EIO).^13^,^14^ Table 2 shows local relapse rates for Out-Trial patients using the ASTRO and ESTRO APBI criteria and for favorable luminal A biology patients.^11^,^15^ The Out-Trial results show low 5-year recurrence rates for ASTRO suitable, ESTRO good, and Luminal A women, suggesting that favorable tumor biology might allow IOERT to obtain acceptable results in patients unsuitable for APBI by ASTRO or ESTRO guidelines.

**Table 2:** Analysis of Out-Trial ELIOT patients by ASTRO and ESTRO Guidelines for APBI

	All	Suitable	Cautionary	Unsuitable	Not accessible
*ASTRO guidelines*					
Patients	1822	295 (16 %)	690 (38 %)	812 (45 %)	25 (1.0 %)
Local relapses	76	3	21	50	2
5-year rate	6.0 %	1.5 %	4.4 %	8.8 %	9.9 %
Luminal A	648 (36 %)	118 (40 %)	271 (39 %)	251 (31 %)	8
Local relapse	8	2	3	3	0
5-year rate	1.7 %	2.3 %	1.6 %	1.6 %	–
*ESTRO guidelines*					
Patients	1822	572 (31 %)	268 (15%)	965 (53 %)	17 (1 %)
Local relapses	76	7	12	56	1
5-year rate	6.0 %	1.9 %	7.1 %	7.8 %	6.6 %
Luminal A	648 (36 %)	206 (36 %)	129 (48 %)	306 (32 %)	8
Local relapse	8	0	2	6	0
5-year rate	1.7 %	0 %	2.5 %	2.4 %	–

Adapted from Leonardi^13^,^14^ and with permission of Springer Science & Business Media^24^

The University of Verona reported only 1 recurrence at a mean follow-up of 46 months in 226 low-risk women treated with 21 Gy to *D*_max_ (about 10 % lower dose than used in the ELIOT Trial).^16^ Updated results, first presented at the San Antonio Breast Cancer Symposium in 2012, showed only 4 recurrences (1.8 %) with a mean follow-up of 51 months.^17^ The median follow-up is now 5 years with no further recurrences.

In ELIOT patients, 14 of 35 (40 %) of the ipsilateral recurrences were “elsewhere” recurrences, raising the question of whether the applicator size might have been too small to adequately treat microscopic disease extending beyond the excised tumor. The ELIOT authors write: “The difficulty (with IOERT APBI) is not only to define patients at low risk of harboring microscopic disease beyond the tumor site, but also to define the proper coverage of the tumor bed.”^5^

 This hints that larger applicators may have reduced recurrence rates, a concern confirmed by Leonardi et al.^13^ Noting the 4-cm median applicator size used in the ELIOT Out-Trial study and that the pattern of recurrences indicated neoplastic tumor foci outside the effective radiation field, she stated that they were planning to increase the field size used in ELIOT. With a 4-cm applicator, despite the IOERT surgical preparation bringing almost 2 cm of surrounding tissue under the applicator, only about 1.5 cm of surrounding tissue is irradiated to the prescription dose of 90 % because electron applicators have cold spots in the field periphery. Krechetov estimates that, depending on the energy, a 4-cm applicator covers at most only 55 % of the clinical treatment volume (“CTV”) to the 90 % prescription dose.^18^ To ensure uniform coverage of microscopic residual disease, the IOERT applicator should have a circumferential dimension at least 1.5 to 2 cm larger than the maximum tumor dimension. The applicator sizes used in the ELIOT Trial are not specified, but the current guidelines for ELIOT at the EIO (Table 3) indicating larger field sizes, as suggested by Leonardi, are now preferred.^13^

**Table 3:** Reported guidelines at the EIO for low-risk IOERT Group

Age	≥60 years
Tumor size	<2 cm
Applicator size	6 cm minimum, 5 cm occasionally
Grade	G1/G2
ER status	ER+
Proliferative index	Ki-67 < 20
Biology	Luminal A
Lobular CA	Only with MRI assessment

As reported at ISIORT 2012, Baveno, Italy, and with permission of Springer Science & Business Media^24^

Patients found with higher risk factors post-IOERT will also receive 8 fractions of 3.6–4.0 Gy of EBRT, excluding the breast volume irradiated by IOERT

In Verona, where applicator size was selected to be approximately 2 cm circumferentially larger than the largest tumor dimension, median applicator size was 6 cm, 87 % were >5 cm, and 31 % were >6 cm, ensuring good coverage of the tumor bed.^17^

The lower dose used in Verona has lower toxicity and results in a higher percentage of the clinical treatment volume receiving the prescription dose. The higher dose used in the ELIOT study has acceptable toxicity at 5 years, but the higher ELIOT dose could impact longer-term cosmesis, especially when larger applicators are used.^12^

In the ELIOT Trial, 53 of 651 (8.1 %) of the IOERT patients had invasive lobular carcinoma (ILC).^5^ ILC is generally excluded in APBI trials. In the ELIOT study, ILC did not surface as an ELIOT high-risk factor, but 5 of 35 (14.3 %) of the total IOERT recurrences were from ILC or mixed IDC/ILC patients. Maluta, comparing his APBI patients with those in the ELIOT Out-Trial, found that ILC might be a risk factor (*p* = .04) in patient selection.^16^,^19^ The current ELIOT policy at the EIO permits ILC only after MRI assessment (Table 3).

Univariate analysis in the ELIOT Out-Trial showed that 3 or more positive nodes was a risk for recurrence, but not a factor in the multivariate analysis.^19^ However, annual rates of recurrence with 3 or more positive nodes compared unfavorably with patients with 0 or 1–2 positive nodes: for true recurrences (1.57 vs 0.69 % or 0.70 %), ipsilateral breast elsewhere recurrences (1.35 % vs 0.29 % or 0.69 %), and annual rates of breast cancer deaths (2.97 vs 0.52 % or 0.70 %).^19^ The University of Verona included 50 patients (22.1 %) that had either a positive SNB at the time of surgery or final pathology who also underwent complete axillary lymph node dissection during the initial surgery or at a second operation.^16^ A total of 38 patients had 1 positive lymph node and 12 had 2. All received IOERT. None of the recurrences at 5 years (Table 4) had a positive sentinel node. In the ELIOT Trial patients were stratified as N0, 1–3 positive nodes, or 4 or more positive nodes.^5^ The presence of 4 or more positive nodes disqualified a patient as ELIOT Low Risk. Patients with 1–3 positive nodes had a 5-year recurrence rate of 5.3 %, and 10 of 35 (28 %) of the recurrences came from this group. It would have been instructive to see if patients with only 1 or 2 positive nodes had a lower recurrence rate as the ELIOT Out-Trial suggests.^19^ It would also allow treatment decisions to be made per the American Society of Breast Surgeons guidelines based on the ACOSOG Z0011 treatment of patients with 1 or 2 positive nodes.^20^,^21^

**Table 4:** University of Verona APBI 5-year IBTR recurrences compared with ASTRO/ESTRO low-risk women

Factor	Patient 1	Patient 2	Patient 3	Patient 4	ASTRO suitable	ESTRO good
Age, years	55	62	68	75	≥60	≥50
Histology	IDC	IDC	IDC	IDC	IDC^b^	IDC^b^
Tumor size	2.0 cm	2.8 cm	1.5 cm	1.8 cm	≤2.0 cm	≤3.0 cm
Nodal status	pN0	pN0	pN0	pN0	pN0	pN0
Grade	G2	G2	G3	G3	Any	Any
ER status	Positive	Positive	Positive	Negative	Positive	Any
PR status	Positive	Positive	Positive	Negative	Positive	Any
HER2- status	Negative	Unknown	Negative	Unknown	NS	NS
Margin status	Negative	Negative	Negative	Negative	Negative	Negative
Adjuvant HT/CT	HT	HT	NT	None^a^		
Time to relapse	28 months	36 months	40 months	60 months		
Salvage	Mastectomy	Mastectomy	Mastectomy	Mastectomy		

Adapted from Maluta^17^ and with permission of Springer Science & Business media^24^

^a^No adjuvant treatment due to age. Note that 3 of 4 recurrences did not meet ASTRO suitable guidelines for APBI, while all but the triple negative patient met the ESTRO good guidelines for APBI. If G3 and tumors > 2.0 cm are excluded, there is only 1 recurrence in the University of Verona cohort with a median follow-up now of 5 years

^b^Other low-risk histologies are permitted. All patients are still alive at 5 years

Some IOERT APBI centers perform definitive node assessment in a separate out-patient surgery prior to tumor removal, and only patients with pN0 receive IOERT APBI. While this resolves nodal status, it subjects all patients to a second surgical procedure that 70–75 % will not require.

Margins are not discussed, but we know from previous ELIOT presentations that the positive margin rate was very low: only 3 (0.5 %) in the ELIOT arm and 9 (1.4 %) in the EBRT arm.^22^ Other centers treating with IOERT APBI that find positive margins either re-excise or ignore them, with no apparent impact on recurrence reported to date. In the Maluta study, none of the 16 patients (7.1 %) with positive margins or the 17 patients (7.5 %) with close margins have recurred.^16^ Jobsen has demonstrated that margin positivity in older women does not seem to impact recurrence.^23^ More data are needed to determine the impact of positive margins on recurrence.

APBI requires proper patient selection and proper technical implementation. The ELIOT Trial and other APBI IOERT published studies show a probable subset of women who can safely benefit from this 1-day treatment. These are the ELIOT Low Risk, ASTRO suitable, ESTRO good, or Luminal A patients. The current guidelines for ELIOT at the EIO are shown in Table 3.

The ELIOT authors suggest that preoperative criteria (e.g., tumor size, age, and pathological and biological examination of the biopsy specimen) can be used to identify suitable patients. A second option, they say, would be to treat all patients with IOERT, and after postsurgical categorization, give WBI to patients at high risk for recurrence. However, this is technically challenging, as the WBI should avoid overlap with the volume of tissue irradiated with IOERT. The better solution is to select APBI patients who are suitable for IOERT and who can then be expected to have low 5-year recurrence rates with no adverse impact on overall survival.

## ELIOT Conclusions

The ELIOT trial has contributed to our understanding of whether a single-dose treatment using electrons may be possible. The Trial included some high-risk patients that today would not be considered a good choice for APBI. It appears, however, that IOERT APBI may have a subset of low-risk women (ASTRO suitable, ELIOT Low Risk, Luminal A) for whom IOERT could be effective, with a recurrence rate in the 2 % range at 5 years. In spite of a 5.8-year median follow-up, the ELIOT data are still early and single-fraction IOERT patients should be treated under strict institutional protocols. When long-term results are available, it is likely there will be a higher overall recurrence rate for IOERT when compared with EBRT, but we should be able to select subgroups of favorable patients where this difference is small and acceptable. How much additional risk of local recurrence is acceptable will vary with patients and the situation in which they find themselves. Overall, the results of the ELIOT Trial are reasonably mature and encouraging.
